# The BeachLitter dataset for image segmentation of beach litter

**DOI:** 10.1016/j.dib.2022.108072

**Published:** 2022-03-22

**Authors:** Daisuke Sugiyama, Mitsuko Hidaka, Daisuke Matsuoka, Koshiro Murakami, Shin'ichiro Kako

**Affiliations:** a Research Institute for Value-Added-Information Generation (VAiG), Japan Agency for Marine-Earth Science and Technology (JAMSTEC), 3173-25 Showa-machi, Kanazawa-ku, Yokohama, Kanagawa 236-0001, Japan; b Department of Engineering, Graduate School of Science and Engineering, Ocean Civil Engineering Program, Kagoshima University, Japan

**Keywords:** Beach litter, Marine plastics, AI, Deep learning, Image segmentation, Beach monitoring

## Abstract

This dataset consists of 3500 images of beach litter and 3500 corresponding pixel-wise labelled images. Although performing such pixel-by-pixel semantic masking is expensive, it allows us to build machine-learning models that can perform more sophisticated automated visual processing. We believe this dataset may be of significance to the scientific communities concerned with marine pollution and computer vision, as this dataset can be used for benchmarking in the tasks involving the evaluation of marine pollution with various machine learning models. The beach litter images were obtained from coastal environment surveys conducted between 2011 and 2019 by the Yamagata Prefectural Government, Japan. These images were originally obtained owing to the reporting guidelines concerning regular coastal-environmental-cleanup and beach-litter-monitoring surveys. Based on these images, the Japan Agency for Marine-Earth Science and Technology created 3500 images comprising eight classes of semantic masks for beach litter detection [1].

## Specifications Table

**Table:** 

Subject	Computer Vision and Pattern Recognition
Specific subject area	Pixel-level image classification for detection of beach litter
Type of data	JPEG images, PNG images
How the data were acquired	The original images had been captured by various observers using standard consumer digital cameras. Image resolutions and JPEG data compression ratios are not consistent across different digital cameras. Therefore, Python 3.8 and Pillow 7.2 were used to create resolution-normalized versions of these images. Subsequently, segmentation masks were created by placing polygons over the original images using the Labelme annotation tool (version 4) [2].
Data format	Raw, Annotated
Description of data collection	To develop a monitoring method for the evaluation of marine pollution, we collected images of beach litter on sand beach. The original images were captured in terms of the following four directions: the front (facing the sea), the right side, the left side, and the back from the same shooting point.
Data source location	Institution: Yamagata Prefecture Government City/Town/Region: Yamagata Prefecture Country: Japan Images of all the sand beaches in the Yamagata Prefecture were utilized.
Data accessibility	Repository name: SEANOE - Sea Open Scientific Data Publication Data identification number: 85472 Direct URL to data: https://doi.org/10.17882/85472
Related research article	M. Hidaka, D. Matsuoka, D. Sugiyama, K. Murakami, S. Kako, Pixel-level image classification for detecting beach litter using a deep learning approach, Marine Pollution Bulletin 175 (2022).

## Value of the Data

• Because manually generating high-quality pixel-by-pixel annotations is more expensive than creating annotations for image classification or object detection, such annotations are rare. In particular, creating annotations for beach litter images, which involve many scattered objects, is technically complex and expensive. To annotate this dataset, 3500 images were annotated by 15 annotators over an approximately two-month period. Three quality controllers and one researcher checked the annotation quality.
• The beach litter images were obtained according to the guidelines in (Non-Profit Organization Partnership Office, 2021), and they provide valuable data through long-term observations collected over an eight-year period from 2011 to 2019.
• This dataset allows researchers to benchmark various tasks involved in beach pollution problems, for example, classification of artificial or natural litter such as marine plastics in terms of pixels and the detection of landscape types such as sky, sea, and sand. This dataset is available to discuss and compare these tasks: e.g., estimating volume or area of beach litter.

## 1 Data Description

**Fig. 1:**
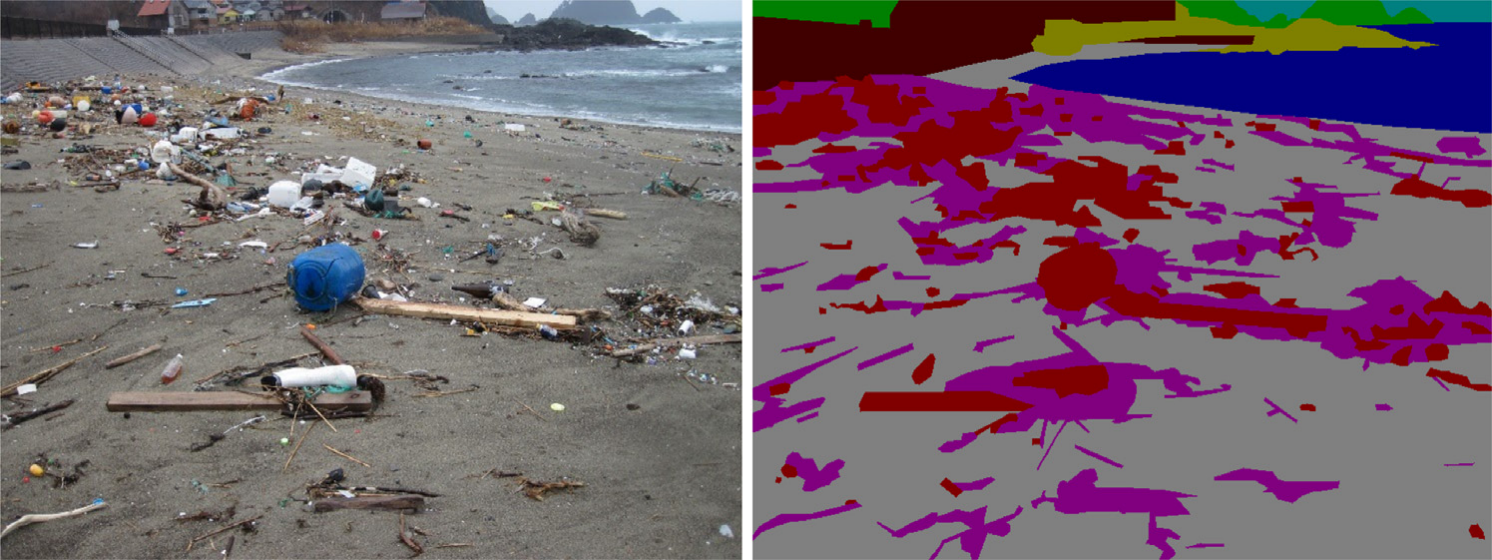
Original image of beach litter (left), and the corresponding 8-class-segmentation mask (right). For color definitions of classes, see Table 2.

The BeachLitter dataset contains 3500 original beach litter images and manually annotated segmentation masks divided into eight classes: artificial litter, natural litter, sandy beach, sea, sky, living, non-living, and background (Fig. 1). For a similar data format of image segmentation, refer to [4].

The BeachLitter dataset is provided by the BeachLitter directory, which contains the original images, and the for_paper_MPB2022 directory, as used in [1]. For the directory tree and description of this dataset, see Table 1.

**Table 1:** Overview of the dataset directory structure and files.

Directory tree	Description
├— BeachLitter/	
│ ├— images/	Directory of original beach litter JPEG images.
│ ├— maskpngs/	Directory of annotated PNG images.
│ └— labels.txt	Text file containing class definitions.
├— for_paper_MPB2022/	
│ ├— train.lst	List of training sets.
│ ├— test.lst	List of test sets.
└— README	Links for paper, disclaimer, etc.

**Fig. 2:**
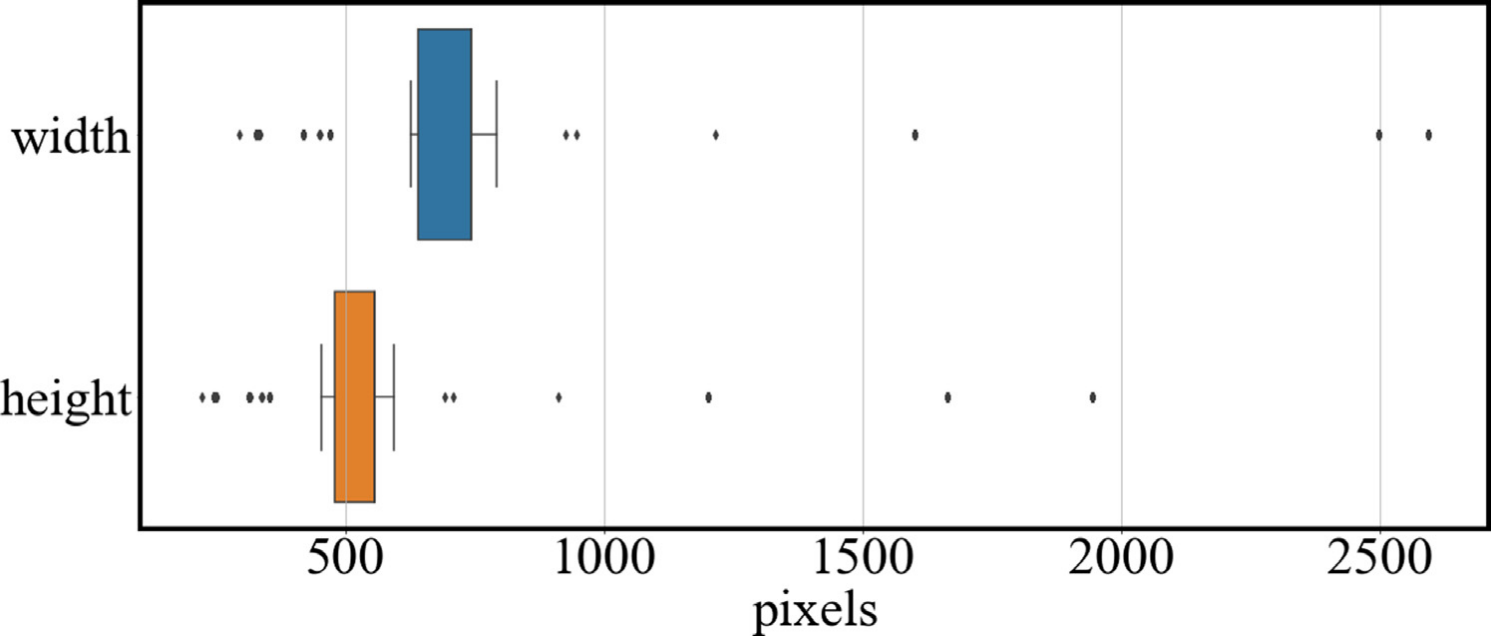
Boxplot of width and height of the original images. Both ends of the box are quartiles; the lines are at 1.5 times the quartile range, and the black dots represent outliers of this range.

**Fig. 3:**
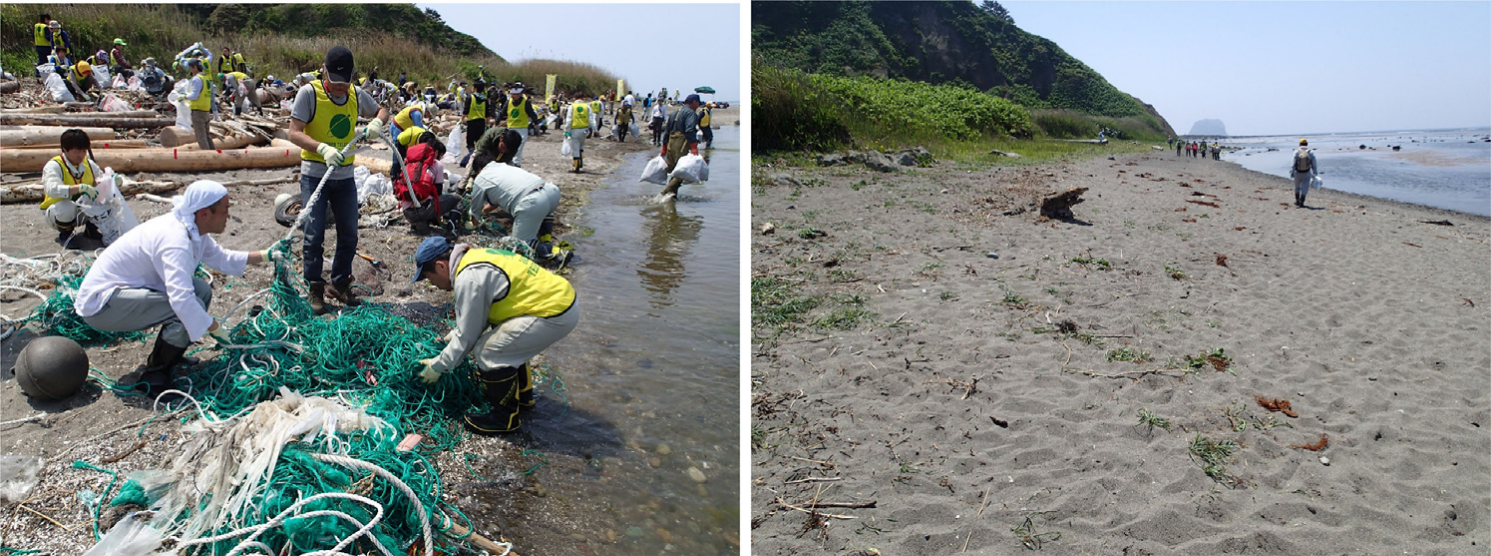
(Left) Cleanup event at Tanoshiri beach by the Non-Profit Organization Partnership Office. (Right) The same beach after the cleanup. Before the cleanup, surveyors dispatched by Yamagata Prefecture conducted a preliminary survey by taking pictures with a digital camera.

BeachLitter directory:

The images directory contained the original JPEG images, and the corresponding segmentation-mask PNG images were present in the maskpngs directory. The 3500 image pairs were named using corresponding numbers, for example, 001234.jpg and 001234.png. The resolutions, aspect ratios, and compression ratios of different JPEG images were not consistent. The statistics concerning image resolutions are shown in Fig. 2. The images were saved at various resolutions: the median width was 773 px, while the median height was 557 px. The aspect ratio of 95.5% of the images was 4:3. Eighty four percent of the JPEG images did not possess an exchangeable image file format tag; therefore, the compression ratios of these images was not clear. These characteristics are due to the fact that these images were captured by various observers using different cameras over a long period from 2011, during beach cleanup and monitoring event (Fig. 3).

The PNG images in the maskpngs directory were indexed using the PNG-8 format. Indexed color is a PNG format that records an image by arbitrarily selecting colors and assigning integer index numbers between 0 to 255. The color legend and index color assignments are listed in Table 2. For using the segmentation masks, users can read the PNG index code to determine the class or match RGB color values programmatically. Additionally, labels.txt is a text file containing these class definitions. for_paper_MPB2022 directory:

**Table 2:** PNG index codes and RGB values of the colors of each segmentation-mask class in the maskpngs directory.

This directory contains the train.lst and test.lst files, which correspond to the training and test sets used in [1], respectively.

## 2 Experimental Design, Materials and Methods

Coastal authorities of Yamagata Prefecture have been making efforts to clean beaches to make them safe for people to walk barefoot (Fig. 3). To determine the number of people needed for cleanup, it is necessary to know the conditions of the beaches to be cleaned. To aid this process, beach pollution has been monitored at periodic intervals since 2011.

The Non-Profit Organization Partnership Office has developed a visual observation method for monitoring coastal litter to compare the cleanliness of beaches in a uniform manner (see details in [3]). Yamagata Prefecture has been monitoring coastal litter using this method twice every year in spring and autumn. This method considers three different conditions of the beaches as Cases A, B, and C. Case A indicates a sandy beach that is somewhat wide to the wash zone; in this case, observers note the four directions front, left, right, and back of the sea to determine the cleanliness rank. Case B indicates a sandy beach with a shallow depth to the wash zone, and in this case, the observers refer to three directions: front, left, and right. Case C indicates a rocky shore with a shallow depth to the wash zone, and in this case, observers refer to the same three directions: front, left, and right. Photographs were captured during the monitoring process based on these three different cases and corresponding rules. We used photographs from Case A for constructing the dataset.

We extracted the images from the reports of these observations (Excel files) and filtered them using Case A. Subsequently, we obtained approximately 7000 images. However, because we had to annotate the images under a limited budget, only 3500 images were randomly sampled from this set. Thereafter, the segmentation masks of these images were created by 15 annotators over a period of approximately two months. Eight segmentation classes were defined for making the masks: Sky, sea, sand beach, artificial litter, natural litter, living, non-living, and background. Artificial litter indicates the litter caused by human activity, and natural litter indicates that the litter originated in nature.  Definitions of the artificial and natural classes are based on the local governmental coastal litter monitoring concept in Yamagata prefecture, Japan. Other classes were defined to distinguish from litter classes and those were also necessary to estimate the area covered by litter. Labelme (version 4) [2] was used as the semantic annotation tool by the annotators. The annotators visually checked the coast image and manually connected the points to create the target object area using this software. Automated verification of pixels of leftover paint could be performed; however, it was very difficult to identify and correct instances of misclassification. Therefore, the quality of annotated images was verified by three quality-controllers and one marine science researcher. The quality control of segmentation masks took approximately one month. Finally, this dataset was subsequently image-preprocessed, normalizing the image-size (see details in [1]), to enable a variety of experiments to be performed for training or analyzing results. However, since the suitable image size varies depending on each machine learning model, this dataset contained the raw images before resizing.

## Ethics Statements

Not applicable.

## CRediT authorship contribution statement

**Daisuke Sugiyama:** Conceptualization, Data curation, Methodology, Formal analysis, Software, Writing – original draft. **Mitsuko Hidaka:** Investigation, Data curation, Software, Writing – review & editing. **Daisuke Matsuoka:** Project administration, Formal analysis, Writing – review & editing. **Koshiro Murakami:** Formal analysis, Software. **Shin'ichiro Kako:** Writing – review & editing.

## Declaration of Competing Interest

To the best of our knowledge, the named authors have no conflict of interest, financial or otherwise.

## Data Availability

The BeachLitter Dataset v2022 (Original data) (Seanoe).
